# Transfer RNA and human disease

**DOI:** 10.3389/fgene.2014.00158

**Published:** 2014-06-03

**Authors:** Jamie A. Abbott, Christopher S. Francklyn, Susan M. Robey-Bond

**Affiliations:** Department of Biochemistry, College of Medicine, University of VermontBurlington, VT, USA

**Keywords:** tRNA, neurodegenerative disease, localized translation, mitochondrial disease, aminoacyl-tRNA synthetase, Usher syndrome Type IIIB

## Abstract

Pathological mutations in tRNA genes and tRNA processing enzymes are numerous and result in very complicated clinical phenotypes. Mitochondrial tRNA (mt-tRNA) genes are “hotspots” for pathological mutations and over 200 mt-tRNA mutations have been linked to various disease states. Often these mutations prevent tRNA aminoacylation. Disrupting this primary function affects protein synthesis and the expression, folding, and function of oxidative phosphorylation enzymes. Mitochondrial tRNA mutations manifest in a wide panoply of diseases related to cellular energetics, including COX deficiency (cytochrome C oxidase), mitochondrial myopathy, MERRF (Myoclonic Epilepsy with Ragged Red Fibers), and MELAS (mitochondrial encephalomyopathy, lactic acidosis, and stroke-like episodes). Diseases caused by mt-tRNA mutations can also affect very specific tissue types, as in the case of neurosensory non-syndromic hearing loss and pigmentary retinopathy, diabetes mellitus, and hypertrophic cardiomyopathy. Importantly, mitochondrial heteroplasmy plays a role in disease severity and age of onset as well. Not surprisingly, mutations in enzymes that modify cytoplasmic and mitochondrial tRNAs are also linked to a diverse range of clinical phenotypes. In addition to compromised aminoacylation of the tRNAs, mutated modifying enzymes can also impact tRNA expression and abundance, tRNA modifications, tRNA folding, and even tRNA maturation (e.g., splicing). Some of these pathological mutations in tRNAs and processing enzymes are likely to affect non-canonical tRNA functions, and contribute to the diseases without significantly impacting on translation. This chapter will review recent literature on the relation of mitochondrial and cytoplasmic tRNA, and enzymes that process tRNAs, to human disease. We explore the mechanisms involved in the clinical presentation of these various diseases with an emphasis on neurological disease.

## Introduction

Although the role of tRNA in translation has been known since the late 1950's, the first report linking a mutation in tRNA to a human disease was published in 1990, when MELAS was associated with a mutation in the mitochondrial tRNA^Leu^ gene [mitochondrial tRNA Leu (L)] (*MTTL1*) (Kobayashi et al., 1990). This report was followed by the demonstration that tRNA^Lys^ is associated with MERRF (Shoffner et al., 1990). Since then, multiple mutations in individual tRNA genes have been associated with multiple diseases, and individual diseases have been found to be caused by mutations in one of several tRNAs. Not unexpectedly, the penetrance and severity of disease caused by tRNA mutations has been demonstrated to be unpredictable (Phizicky and Hopper, 2010; Hurto, 2011; Suzuki et al., 2011; Tuller, 2012). The primary role of tRNA is to deliver amino acids to the nascent polypeptide chain during protein translation, a seemingly irreplaceable function. Disease-causing mutations in tRNA, to date, have been found only in mitochondrial tRNA, indicating that the etiology of tRNA-linked diseases are tightly associated with mitochondrial biology. How can different point mutations in the same molecule lead to very different diseases? Do tRNAs play additional roles in mitochondria or in the cell apart from translation, for example in organelle localization or replication, cell death, membrane environment, or DNA replication (Dimauro et al., 2013)? About 1700 mitochondrial proteins are coded in the nuclear genome, and it is unknown how mutated mt-tRNA might interact with these.

Many macromolecules interact with tRNA in the cytosol as well. The association of mutations in tRNA binding or processing enzymes with disease is even more recent: tRNA splicing endonuclease mutations were found to cause pontocerebellar hypoplasia as recently as 2008 (Budde et al., 2008). Mutations in the canonical tRNA partner, aminoacyl tRNA-synthetases (ARSs), were first identified as causative agents of Charcot-Marie-Tooth in 2003 (Antonellis et al., 2003). The subject of ARSs and diseases has also received attention, with a number of useful recent reviews addressing various issues (Kim et al., 2011; Guo and Schimmel, 2013; Jia et al., 2013; Konovalova and Tyynismaa, 2013; Schwenzer et al., 2013; Diodato et al., 2014). A major question yet to be addressed is the localization of translation in neural cells, and how tRNA may be trafficked to ribosomes in distant regions (e.g., long axons and synapses in neurons) of the cell.

In this review, we survey the known and emerging connections between tRNA and human diseases, and the role of its key cellular partners in pathophysiology. We begin in the mitochondria, as only mitochondrial tRNA mutations have been found, and briefly review how mitochondrial biology contributes to the effects of tRNA mutations. Several tissue-specific diseases are reviewed, followed by a discussion of potential therapeutics. Next, we move into the cytoplasm to investigate tRNA interactions with other molecules—specifically modifiers and splicing endonucleases, followed by the role tRNA binding plays in regulating protein synthesis. Finally we review the canonical role of tRNA interactions with aminoacyl synthetases, with an emphasis on recent disease discoveries.

## Mitochondrial tRNA mutations and disease

### Mitochondrial functional biology and genomics

Mitochondria perform the essential function of synthesizing ATP in eukaryotic cells, and this cellular energy resource powers the biosynthesis of key metabolites, mechanochemical and transport functions, and a vast array of other activities. In addition to these functional roles, the mitochondria also generate and regulate the production of reactive oxygen species, as well as activate important cellular pathways such as apoptosis. The 16.59 kb double stranded circular mitochondrial genome is located within the inner mitochondrial membrane, and encodes for a total of 37 genes. Thirteen of these encode for open reading frames that comprise most of the subunits of the respiratory chain complexes I (seven subunits), III (one subunit), IV (three subunits), and V (two subunits). The remaining subunits of the respiratory chain complex are encoded by genes located in the nuclear genome. Two mitochondrial genes encode for ribosomal RNAs (rRNAs) 16S and 12S and the remaining 22 genes encode for the set of mitochondrial transfer RNAs (mt-tRNA). All of the RNA molecules necessary for mitochondrial translation are provided for by the mitochondrial genome. However, many proteins necessary to perform protein synthesis, including mitochondrial aminoacyl tRNA synthetases (mt-ARS), ribosomal proteins, and tRNA processing enzymes are encoded in the nuclear genome. These are synthesized in the cytoplasm and subsequently imported into the mitochondria.

### Mitochondrial tRNA mutations and disease

It is well established that many different mt-tRNA mutations can cause a wide range of human diseases. High-energy consuming tissues such as muscular and nervous systems are particularly affected by mitochondrial defects. Unlike chromosomal DNA localized to the nucleus and subject to Mendelian inheritance, mitochondrial DNA is inherited solely from the mother and thousands of identical copies can exist per cell. Cells that carry a homogeneous population of the mitochondrial genome, wild type or mutated, are referred to as homoplasmic. A condition in which more than one type of mitochondrial genome exists, either in a cell, tissue or in an individual, is referred to as heteroplasmy. Should a mutation spontaneously arise or be maternally inherited, mitochondria carrying the pathogenic mutated mtDNA can accumulate over the course of organismal development. When the mutational load of a certain tissue type reaches a particular threshold, disease symptoms can become evident. Depending on the mutation and the tissue, this threshold can vary from 50 to 90%. Mitochondrial diseases and their respective tRNA mutations have been extensively cataloged, and the information is available in a variety of useful databases (Putz et al., 2007; Ruiz-Pesini et al., 2007). Past and recent examples of mt-tRNA disease-causing mutations demonstrate that these tRNA genes can affect mitochondrial protein synthesis, aminoacylation activity, and three-dimensional structure and folding of the tRNA in the mitochondria (Table 1). Mitochondrial tRNA structures can differ significantly from cytosolic tRNA (Florentz et al., 2003) and many require specific complex modifications in order to fold correctly. Here we will review some well-characterized and recently reported mt-tRNA mutations, particularly as they relate to neurobiology and specific human disease phenotypes.

**Table 1:** **Mitochondrial tRNA mutations and disease**.

**Mt-tRNA gene**	**Mutation**	**Disease**	**Structural location**	**Aberrant tRNA biology**	**OMIM#**	**References**
*MTTL1*	A3243G	MELAS	anti-codon (wobble position WP)	defect in taurine modification	540000	Latkany et al., 1999
*MTTK*	A8344G	MERRF	anti-codon (WP)	defect in taurine modification	545000	Shoffner et al., 1990
*MTTI*	C4277T	CMH1	DUH stem	reduced expression in cardiac tissue	192600	Taylor et al., 2003
	A4300G		Anticodon stem			Perli et al., 2012
	G4308A	CPEO	TΨC stem	misfolding leads to improper 3′ end processing	590045	Schaller et al., 2011; Souilem et al., 2011
	A4302G		Variable loop	disrupt conserved base pairing		Berardo et al., 2010
*MTTH*	G12192	CM	TΨC stem	disrupt conserved base pairing	590040	Shin et al., 2000
	G12183A	NSHL	TΨC stem	disrupt conserved base pairing	500008	Crimi et al., 2003
		RP				
	T12201C	NSHL	Acceptor stem	reduced expression of functional tRNA		Yan et al., 2011
	A12146G	MELAS	DHU stem	misfolding	540000	Calvaruso et al., 2011
	G12147A	MELAS	DHU stem	misfolding and low abundance	540000	Melone et al., 2004
		MERRF			545000	
*MTTE*	G14685A	C,SP,A	TΨC stem	disrupt conserved base pairing	590025	Lax et al., 2013

*Disease abbreviations: MELAS, mitochondrial encephalomyopathy, lactic acidosis, and stroke-like episodes; MERRF, myoclonic epilepsy with ragged red fibers; CMH1, cardiomyopathy familial hypertrophic; CPEO, Chronic progressive external ophthalmoplegia; CM, cardiomyopathy; NSHL, non-sensory hearing loss; RP, Retinitis pigmentosa; C,SP,A, cataracts, spastic paraparesis, and ataxia*.

### MELAS and MERRF

The two classic well-characterized diseases associated with mt-tRNA mutations are mitochondrial encephalomyopathy, lactic acidosis, and stroke-like episodes (MELAS) and myoclonic epilepsy with ragged red fibers (MERRF). These are linked to mutations in mt-tRNA^Leu^ and mt-tRNA^Lys^ respectively (Suzuki et al., 2011). Patients with MELAS present with seizures, recurrent headaches and vomiting, anorexia, exercise intolerance, and proximal limb weakness; these are often seen early in childhood. Recurring stroke-like episodes can progressively impair motor abilities, vision, hearing, and mentation. MELAS is primarily caused by defects in respiratory chain complexes I and IV, which lead to impaired oxidative phosphorylation. The A3243G mutation, which encodes a mutation in the mt-tRNA^Leu^ wobble position, is common among some 80% of MELAS patients (Goto et al., 1990; Kobayashi et al., 1990). This mt-tRNA^Leu^ A3243G mutation was initially confirmed in 5 patients with MELAS syndrome (Kirino et al., 2005). Interestingly, the A8344G mutation associated with MERRF is also located in a wobble base position, in this case in mt-tRNA^Lys^.

The effects of the mt-tRNA^Leu^ A3243G and mt-tRNA^Lys^ A8344G mutations were initially studied in cybrid cell lines (Kirino et al., 2004) followed by further characterization using mass spectrometry. Cybrid cells are created by fusion of cells that are free of mitochondria (rho 0 cells) with donor platelets, or by fusion of enucleated patient derived fibroblasts with osteosarcoma cells that lack mtDNA. These cells thus possess mtDNA from patients or controls in a control nuclear background. Mutations linked to MELAS were subsequently shown to affect the τm^5^U modification for mt-tRNA^Leu^ and the τm^5^s^2^U modification for mt-tRNA^Lys^ at the wobble position in the anticodon region (Yasukawa et al., 2000). These wobble position modifications are both essential for proper decoding by the mitochondrial ribosome (Kirino et al., 2004). Additional details concerning the biochemical affects of these taurine defective mutations have been recently reviewed (Suzuki et al., 2011).

**Figure 1:**
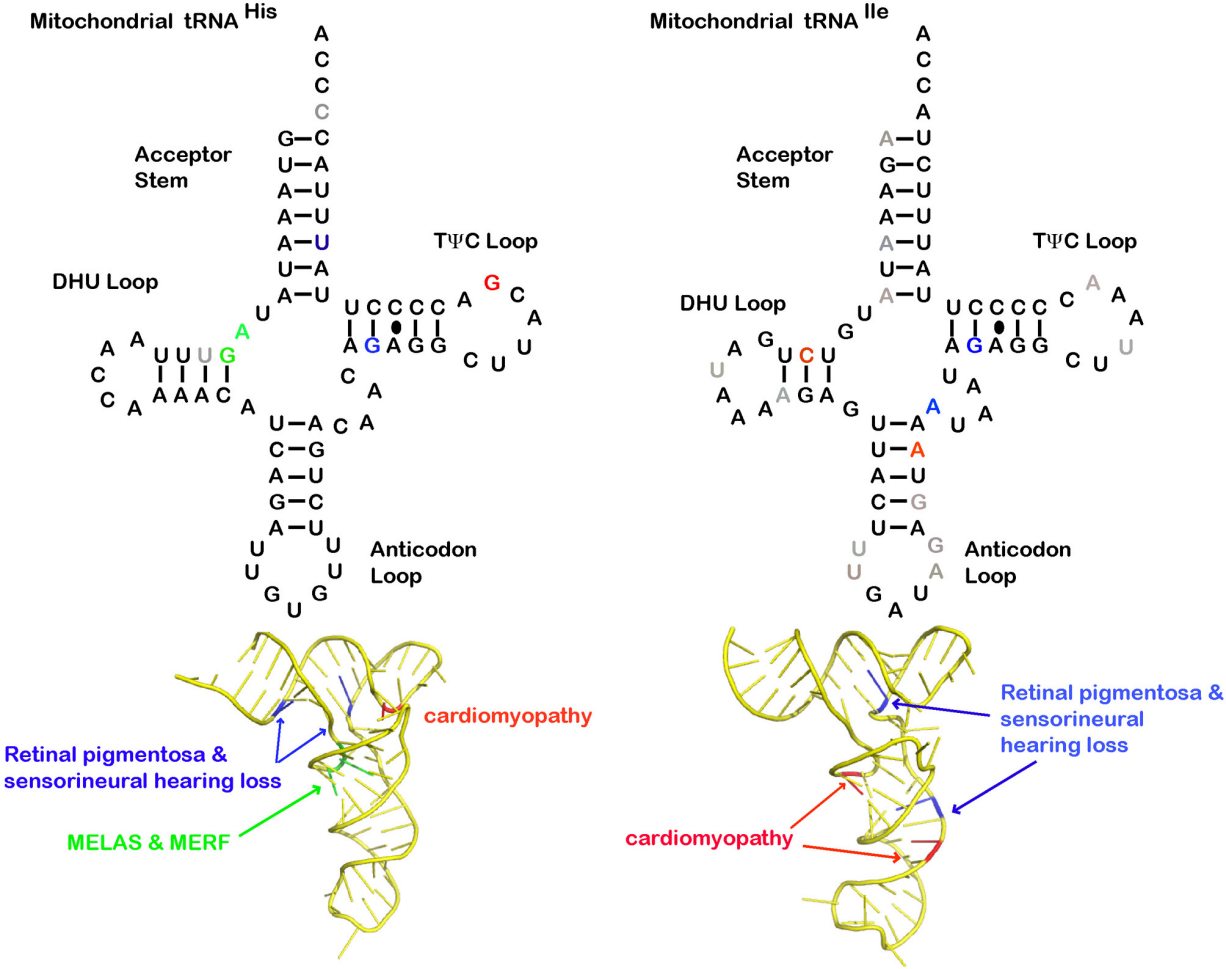
**Structural analysis of mitochondrial tRNA and disease causing mutations**. Cloverleaf structure of mt-tRNA^His^ and mt-tRNA^Ile^. Mutations discussed in this review that cause neurosensory disease (blue), cardiomyopathy (red), MELAS and MERRF (green), and other pathological reported mutations not discussed in this review (gray). The 3D mt-tRNA^His^ and mt-tRNA^Ile^ models were generated using ModeRNA (Rother et al., 2011) of human mt-tRNA sequence (Sprinzl and Vassilenko, 2005) alignments. Sequences were fit to structural tRNA templates PDB: 1QTQ for mt-tRNA^His^ model and PDB: 1QUZ for mt-tRNA^Ile^.

Other mt-tRNA mutations associated with MELAS and MERRF phenotypes have been isolated in the gene encoding mt-tRNA^His^ (Figure 1). In the first reports, the mutation was found to be a heteroplasmic G12147A substitution (Melone et al., 2004; Taylor et al., 2004), and analysis of muscle biopsy samples revealed deficiency in cytochrome *c* oxidase (COX). The G12147A mutation in the D-arm of the mt-tRNA^His^ molecule would be expected to alter tRNA folding and abundance, leading to a specific decrease in the his-rich COXIII polypeptide, as well as a general decrease in mitochondrial protein synthesis. Additionally, a homoplasmic A12146G mutation was identified in another MELAS patient (Calvaruso et al., 2011). As in the previous example, an analysis of cybrid cells and patient muscle biopsy samples showed that deficiencies in various respiratory chain complexes enzymes were linked to the mt-tRNA^His^ mutation.

### Mitochondrial biology and tissue specificity of disease

Diseases related to mitochondrial tRNA mutations can manifest in very specific tissue types, leading to complex phenotypes. For example, the heart is heavily reliant on mitochondrial ATP production for proper and synchronized muscle contraction, and is thus particularly sensitive to mt-tRNA mutations. Animal models for heart failure and ischemia/reperfusion frequently display morphological changes to mitochondria that would be expected to exert negative impacts on the function of adult cardiac cells (Piquereau et al., 2013). One such tRNA-linked cardiomyopathy is the homoplasmic G12192A mt-tRNA^His^ mutation localized to the loop of the TΨC arm, which was initially identified in five patients (Figure 1) (Shin et al., 2000). Electron microscopy imaging carried out on patient cardiomyocytes showed a loss of sarcomere formation and an accumulation of mitochondria with aberrant morphology.

Mutations associated with hypertrophic cardiomyopathy have also been found in mt-tRNA^Ile^ (Figure 1) (Taylor et al., 2003; Hollingsworth et al., 2012). For patients carrying these A4300G and C4277T mt-tRNA^Ile^ mutations, the only symptom was hypertrophic cardiomyopathy. However, detailed immunohistochemistry and biochemical studies on the hearts of patients after transplant surgery indicated a large proportion of COX-deficient cardiomyocytes and defects in activity for respiratory chain complexes I, III, and IV (Giordano et al., 2013). Also, there appeared to be reduced expression of mt-tRNA^Ile^ in both left and right ventricle samples from two patients compared with control mt-tRNA^Leu^. This suggests that the phenotype may arise from an increasing number of COX-deficient cardiomyocytes; however, the numbers of COX-deficient cells in cardiac muscle have been shown to increase with age.

In addition to the classic MELAS and MERRF cases, mutations in other mt-tRNAs are also linked to sensorineural defects. A number of reports suggest that inner ear hair cells and their connecting neuronal circuitry may be specifically affected by mt-tRNA mutations. In one report, a late onset hearing impairment in a Chinese family was linked to a T12201C mutation in the acceptor arm of mt-tRNA^His^, with the severity of hearing impairment being linked to the degree of heteroplasmy (Figure 1) (Yan et al., 2011). For this mutation, cellular and biochemical analysis point to reduction in the levels of mt-tRNA^His^ as being the source of reduced mitochondrial translation and subsequent respiratory chain defects. Two related patients carrying a mt-tRNA^His^ inherited heteroplasmic G12183A mutation both developed sensorineural pathology, presenting with visual impairment around the average age of 9 (Crimi et al., 2003). This mt-tRNA^His^ mutation alters a highly conserved base pair in the TΨC arm and would most likely disrupt secondary structure. The older sibling had pigmentary retinopathy, neurosensory deafness, and some muscle atrophy and ataxia, while the younger sibling only experienced both visual and inner ear neurosensory deficits. Interestingly, histochemical analysis of muscle biopsy tissues for (COX/SDH) activity did not convincingly demonstrate a complete COX deficiency.

Like the auditory system, the visual system is similarly sensitive to mutations in mt-tRNA. For example, carriers of G12183A mutation in mt-tRNA^His^ often present with a pigmentary retinopathy that leads to photoreceptor degeneration, pigmentary deposits in the retina, and ultimately progressive loss of vision. Specific muscles responsible for ocular movement are another target in the visual system susceptible to mt-tRNA mutations. Chronic progressive external ophthalmoplegia (CPEO) is a neuromuscular disorder characterized by the loss of extraocular muscle mobility, which results in the inability to move the eyes.

In one case report, CPEO was found to be linked to a G4308A mutation in the T-stem of mt-tRNA^Ile^; this substitution disrupts a conserved GC base pair (Figure 1) (Schaller et al., 2011; Souilem et al., 2011). While there was no evidence of maternal inheritance, patient blood samples were homoplasmic for the wild type mt-tRNA^Ile^, and muscle tissue obtained by biopsy was heteroplasmic. Analysis of the latter tissue indicated a link between the mutation and respiratory chain defects, leading to abnormal mitochondrial morphology. The G4308A substitution leads to a misfolded conformation that is likely to be incompatible with 3′ end processing by tRNase Z. A G4302A mt-tRNA^Ile^ mutation located in the variable arm was also found in a small number of CPEO patients (Berardo et al., 2010). Despite a predicted disruption of a conserved base pair in mt-tRNA^Ile^, a muscle biopsy showed normal function of all of the respiratory chain complex proteins (Berardo et al., 2010). Of 22 pathogenic mutations observed in mt-tRNA^Ile^, nine cause CPEO, suggesting that mt-tRNA^Ile^ is a “hot-spot” for CPEO. Most reported CPEO cases involving MTTI mutations are heteroplasmic and restricted to skeletal muscle. MTTI mutations exist that are primarily homoplasmic and can be found in various tissue types. The molecular rationale for the tight linkage between mt-tRNA^Ile^ and CPEO provides a fascinating research question for future study.

In some systems, a tRNA-linked disorder that is primarily a sensory defect may exert negative effects on additional brain and motor neuron functions. One recent study reported that the pathogenic G14685A mutation in mt-tRNA^Glu^ affects both visual and hearing systems (Lax et al., 2013). In this case, the 7 year old affected subject suffered from cataracts, peripheral retinal degeneration, and pigmentary retinopathy. This was followed by bilateral sensorineural hearing impairment as an adolescent. By the age of 40, the patient developed symptoms of spastic paraparesis, ataxia, slurred speech, and incontinence. Subsequent immunohistochemical (IHC) and biochemical analysis of muscle and brain tissues collected post mortem indicated respiratory chain complex I deficiencies. Additional evidence suggested that this heteroplasmic mutation arose sporadically, with brain regions exhibiting the highest degree of heteroplasmy. The mutation is located in the TΨC arm and substitutes a conserved G-C Watson-Crick base pair; detailed functional studies have yet to be conducted. To date there are 12 mutations in the *MTTE* gene encoding mt-tRNA^Glu^ that cause mitochondrial pathologies with very different phenotypes, such as encephalomyopathy, retinopathy, MELAS, and Leber's hereditary optic neuropathy (LHON).

### Mitochondria in neurobiology and additional considerations for mitochondrial disease phenotypes

While most cells are roughly spherical structures with diameters in the range of micrometers to tens of micrometers, neurons possess irregular structures with long dendrites and axons that extend out from the soma at distances from millimeters to a meter. The synapse has the highest energy consumption of the neuron, and mitochondria are responsible for meeting this major energy demand. Among the important ATP dependent processes occurring at the synapse are vesicle exocytosis and endocytosis, maintenance of membrane potential by ion channels, and actin rearrangements of the cytoskeleton. Accordingly, synaptic mitochondria may be docked or anchored in these specialized compartments in order to execute these essential functions (Court and Coleman, 2012; Sheng and Cai, 2012). Owing to their ability to be transported along filamentous structures in the cell, mitochondria are motile and can be readily localized at the synapses and other high demand areas. Additionally, mitochondria are dynamic and can undergo major structural changes such as fusion, fission, and fragmentation.

Neuronal synapses are rich in mitochondria, both at the presynaptic and postsynaptic nerve terminals, and recent work suggests that there may be differences between synaptic vs non-synaptic mitochondria (Gillingwater and Wishart, 2013). In one model, specialized synaptic mitochondria may be synthesized at the soma and then trafficked to the synapse, where they adapt to the specific demands of this specialized environment. Alternatively, synapse specific mitochondria may be generated, selected for, and then trafficked to the synapse. Other considerations are that the mitochondria at the synapse are “older” and therefore not as efficient or that synaptic mitochondria may be synthesized in axons and would be closer to their destination at the axonal synapse (Amiri and Hollenbeck, 2008; Yarana et al., 2012).

These issues bear on the investigation of the above described neurobiological phenotypes, where defects arising from mt-tRNA mutation or mt-ARS mutations cause losses of mitochondrial function that are concentrated at the synapse (Baloh, 2008). While past work underscores how the altered structure, expression, or lack of tRNA modifications resulting from mt-tRNA mutations may disrupt protein translation (Florentz et al., 2003), the apparent tissue specific phenotype of many of these pathophysiological mutations remains to be elucidated. For diseases that affect basic neurobiologial functions, mtDNA tRNA mutations may affect processes beyond mitochondrial translation, including the mitochondrial inner membrane (MIM) lipid environment, dynamics, maintenance, and replication machinery (Dimauro et al., 2013). Mutations in mitofusin, a protein responsible for mitochondrial fusion and fission, cause the peripheral neuropathy disease Charcot-Marie-Tooth (CMT) type 2A (Zuchner et al., 2004; Kijima et al., 2005). In a zebrafish model, the loss-of-function mutation demonstrated that indeed transport of mitochondria along the axon is disrupted and may be the contributing factor in the CMT2A phenotype (Chapman et al., 2013). The issue of how mitochondrial movement may be regulated, and the role of the other essential factors in that process is discussed in detail elsewhere (Schwarz, 2013). Clearly, how specific mt-tRNA mutations affect biological functions of mitochondria is only beginning to be understood, and will remain a fertile area of research for the foreseeable future.

### Future mitochondrial research strategies and potential therapeutic approaches

Therapeutic progress toward cures for mitochondrial diseases has been slowed by the complex genetics associated with mitochondrial inheritance. For example, a mother with a low degree of heteroplasmy in her mtDNA can transmit a higher level of heteroplasmy to her children, a process referred to as a “mitochondrial bottleneck.” Current efforts to better understand mitochondrial inheritance and the pathophysiology of mt-tRNA mutations are benefitting from both traditional systems like cybrid cells, and from induced pluripotent stem cell (iPScs) technology. iPScs can be challenging to develop if only specific tissues of affected patients carry the mt-tRNA mutation. Mito-mouse models have been generated to recapitulate pathogenic mitochondrial DNA mutations (Inoue et al., 2000). Recently this group of investigators generated a MERRF mito-mouse model incorporating the mt-tRNA^Lys^ G7731A mutation (Shimizu et al., 2014), arguably the first of many such models. The animals generated carried varying levels of the mt-tRNA^Lys^ G7731A mutation, and these levels correlated with the severity of the disease phenotype. Selection of oocytes that carried low amounts of the mt-tRNA^Lys^ G7731A mtDNA mutation produced animals that appeared to be phenotypically normal. This work highlights the promise of this technology in generating future mt-tRNA disease models.

Other therapeutic options under active investigation for mitochondrial diseases include cytoplasmic transfer, gene therapy, or even eliminating dysfunctional mitochondria by altering mitochondrial dynamics to favor quality control mechanisms (Dimauro et al., 2013). In yeast, overexpression of the corresponding mt-ARS provides a rescue mechanism for pathological mt-tRNA mutations (De Luca et al., 2006). In human cells that model MELAS, the mt-tRNA^Leu^ A3243G mutation can be rescued by over expression of mt-LeuRS (Park et al., 2008; Li and Guan, 2010). Furthermore, in yeast models of some mt-tRNA^Leu^, mt-tRNA^Val^, and mt-tRNA^Ile^ mutations, overexpression of the carboxy-terminal domain of mt-LeuRS is sufficient to rescue the respiratory chain defects (Francisci et al., 2011). In human cybrid cells overexpression of mt-LeuRS, mt-ValRS, and mt-IleRS has been shown to rescue pathogenic mutations in mt-tRNA^Ile^ (Perli et al., 2014). How the mt-LeuRS C-terminal domain is able to specifically rescue the defect associated with the mutant tRNA remains to be determined, but may involve direct interactions with mt-tRNA^Ile^ (Perli et al., 2014).

Mitochondrial gene replacement offers one of the most dramatic routes to treatment of mitochondrial disease. A recent noteworthy experiment featured the transfer of the nuclear genome from a primate oocyte to an enucleated oocyte of another primate containing only mitochondria (Tachibana et al., 2009). The oocytes generated contained the nuclear genome from two parents but mitochondria from the donor; when implanted in pseudopregnant mother they were able to successfully produce healthy rhesus macaque offspring. Not surprisingly, human oocyte mitochondrial genome transfer is under way (Amato et al., 2014), although research in the U.S. likely to lag relative to Europe, owing to U.S. funding restrictions on human embryo research. While this three-parent *in vitro* fertilization shows tremendous promise as means to produce a child without mitochondrial DNA mutations that retains both parents' nuclear genome, the approach does not address curing existing patients with mitochondrial disease, or treating mtDNA mutations that arise spontaneously.

## Cytoplasmic tRNA cellular interactions and disease

### Disease-associated cytoplasmic tRNA mutations are not observed

**Figure 2:**
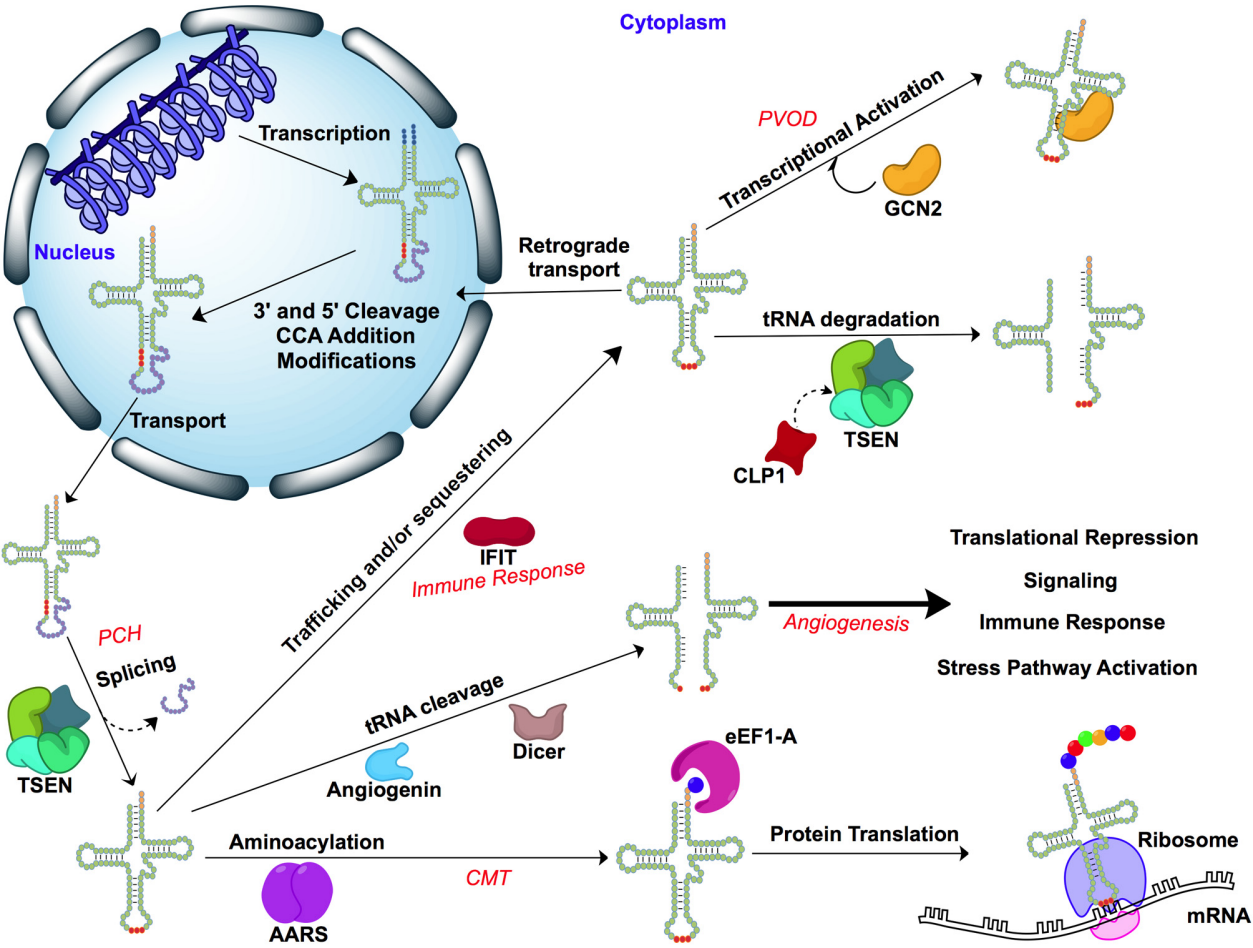
**Transfer RNA interactions and associated diseases**. Cellular pathways of tRNA and interacting proteins and enzymes. Diseases discussed in this review are italicized in red. Figure was generated using BioDraw Ultra 12.0. Disease abbreviations: Charcot-Marie-Tooth disease (CMT), pulmonary veno-occlusive disease (PVOD), pontocerebellar hypoplasia (PCH). Enzyme abbreviations: AARS, aminoacyl-tRNA synthetase; CLP1, cleavage and polyadenylation factor I subunit 1; TSEN, tRNA-splicing endonuclease complex; IFIT, interferon-induced tetratricopeptide repeat; GCN2, general control nonderepressible 2.

The human genome contains approximately 500 tRNA genes, including gene duplications (Lowe and Eddy, 1997; Schattner et al., 2005). However, there are only 61 anticodons specified by the triplet code, so many of these identified tRNA genes share the same anticodon but differ in sequence elsewhere. Remarkably, a human disease that is linked to a mutation in a cytoplasmic tRNA has not yet been reported, and this may be a direct result of the presence of multiple paralogs of the gene encoding each cytoplasmic tRNA molecule. The tRNA for each anticodon, save a single gene for tRNA^Tyr^ (ATA), is encoded by as many as 32 paralogous genes (Chan and Lowe, 2009). The process of generating each mature tRNA is a complex sequence of events that includes gene transcription, splicing, 5′ and 3′ end processing, CCA addition, transportation, and aminoacylation (Hopper et al., 2010). Errors in this sequence arising from mutations in the genes encoding the enzymes that bind and process cytoplasmic tRNA molecules are known, and account for a number of reported diseases. Furthermore, a growing body of literature suggests that tRNA molecules possess previously unrecognized biological functions in eukaryotes which are not fully understood (Phizicky and Hopper, 2010), and that may be perturbed by disruptions of the tRNA-protein interaction. Some of these biological functions include regulation of cellular apoptosis (Mei et al., 2010; Hou and Yang, 2013). See Figure 2 for a schematic overview.

### Role for tRNA modifications in disease

The tRNAs found in higher eukaryotic species are more highly modified relative to prokaryotic and viral tRNA, a distinction that may have arisen from multiple selection pressures. Consequently, various human diseases can arise from improperly modified tRNA molecules (Torres et al., 2014). One element may have been to facilitate immune system discrimination of foreign tRNAs from self-tRNAs (Hanada et al., 2013), discussed below. Modifications of tRNA molecules can serve to support proper folding or facilitate recognition by other interacting enzymes. Classically, impaired modification of mt-tRNA has been associated with reduced recognition by ARS. More recently, methylation of tRNA has been linked to immune response stimulation (or suppression), via toll-like receptor 7 (TLR7) (Kariko et al., 2005; Robbins et al., 2007; Hamm et al., 2010; Jockel et al., 2012). Toll-like receptor activation initiates intracellular signaling events in immune cells by stimulating the release of cytokine type I interferon (IFN). Immune cell TLR7 stimulation can also occur when either viral or bacterial single-stranded RNA or siRNA binds to the receptor in the endosome or cytoplasm (Blasius and Beutler, 2010) and results in IFN production (Takeuchi and Akira, 2010). Purified tRNA from various bacterial species was used to test the immunostimulatory potential in human peripheral blood mononucleated cells (PBMCs). Interestingly, tRNA purified from *T.thermophilus* and *E.coli* did not stimulate IFN-α production from PBMCs. Differences in specific tRNA methylation sites between bacterial species may thus be responsible for loss of TLR7 stimulation and IFN-α production. Isolated tRNA from *E.coli* species lacking a specific tRNA-methyltransferase, the *trmH* encoded Gm18-2'-O-methyltransferase, acquired immunostimulation of TLR7. Curiously, only 7 of 42 tRNAs are Gm18 modified in *E.coli* (Sprinzl and Vassilenko, 2005). Modified Gm18 tRNA mediated inhibition of TLR7 stimulation in mouse FLT3L-induced dendritic cells (DCs) occurs in a dose dependent manner. Also, Gm18-modified tRNA can inhibit viral influenza a virus (IAV) TLR7 stimulation. These experiments demonstrate that Gm18-modified tRNA can act as an antagonist against TLR7-mediated production of IFN-α. In a similar investigation, the 2′-O-methylated Gm18 of yeast tRNA^Phe^ and tRNA^Tyr^ was also found to exert a suppressive affect on the immune response via TLR7 antagonism (Gehrig et al., 2012).

### tRNA splicing and disease

After transcription by polymerase III, many pre-tRNA molecules undergo post-transcriptional alterations to produce a mature product that can be utilized for translation. Initial transcripts are cleaved by the nucleases tRNaseZ and RNase P, which act to form the 3′ and 5′ ends, respectively. Additional modifications are made in the nucleus before transportation into the cytoplasm where, if necessary, alternative splicing can occur. Overall, RNA editing and alternative splicing is highest in the brain compared with other tissues (Norris and Calarco, 2012; Tariq and Jantsch, 2012). In humans the spleen has the highest expression of tRNA followed by the brain (Dittmar et al., 2006). As tRNA splicing has an important role in the central nervous system (CNS) it is not surprising that mutations in the splicing machinery can cause specific CNS diseases. In the human genome there are 32 intron-containing tRNAs (Lowe and Eddy, 1997).

Pontocerebellar hypoplasia (PCH) is a heterogeneous neurodegenerative disorder characterized by defective growth, development, and function of the brainstem and cerebellum. Symptoms of PCH include microcephaly, seizures, hypotonia, hyper-reflexia, and optic atrophy. Magnetic resonance imaging (MRI) of patient brain tissue shows immature development of the cerebellum as well as mild structural defects (Budde et al., 2008). Mutations in the tRNA-splicing endonuclease complex (TSEN2, TSEN15, TSEN34, and TSEN54) were identified in multiple PCH2 and PCH4 patients. An A307S substitution in TSEN54 that is predicted to be non-catalytic appeared in multiple cases (Budde et al., 2008). Notably, TSEN54 is highly expressed in the pons and cerebellar dentate regions of the developing brain (Budde et al., 2008). Two mutations that encode Y309C and R58W substitutions in the catalytic subunits TSEN2 and TSEN34, respectively, were also identified. As these are unlikely to completely abolish endonuclease activity, altered tRNA splicing may not be the sole cause of the phenotype. Alternatively, these might compromise mRNA 3′ end formation or a function of TSEN54 other than tRNA editing, leading to insufficient protein synthesis of specific brain regions during development.

A mutation in a kinase that associates with the TSEN splicing complex affects tRNA processing and is embryonic lethal. Cleavage and polyadenylation factor I subunit 1 (CLP1) is a mammalian RNA kinase capable of phosphorylating the 5′hydroxyl of double and single stranded RNA and has a role in mRNA 3′-end formation (Weitzer and Martinez, 2007; Ramirez et al., 2008). CLP1 associates with the TSEN splicing complex and a role for CLP1 in tRNA processing has recently been demonstrated (Hanada et al., 2013). A mouse model for a CLP1 mutation that abolishes kinase activity was used to examine the phenotypic outcome. Neonatal death of the CPL1 mutant mice was a consequence of respiratory failure and non-viable mouse pups showed a substantial loss of motor neurons. An accumulation of novel tyrosine tRNA fragments derived from pre-tRNA was observed in the brain, muscle, kidney, heart, and liver, while mature tRNA levels remained normal. Recent initial biochemical characterization of CLP1 as part of the TSEN tRNA splicing complex has demonstrated that ATP is not required for CLP1 binding to the complex but CLP1 ATP hydrolysis is required for proper pre-tRNA cleavage (Hanada et al., 2013). Also, efficient pre-tRNA cleavage is protein phosphorylation dependent.

### tRNA fragments and disease

As shown above, tRNA fragments can be associated with disease (Fu et al., 2009). Angiogenin is a ribonuclease that is essential in blood vessel growth and development (Shapiro and Vallee, 1987) and has a role in angiogenesis (Shapiro and Vallee, 1989). Interestingly, *in vitro* full length tRNAs were shown to be substrates for angiogenin (Saxena et al., 1992). Under a broad range of stresses that include heat shock, hypoxia, hypothermia, and nutritional deprivation stress-induced small RNAs (tiRNAs) of 30–40 nucleotides can be generated by cleavage in the anticodon region (Fu et al., 2009). Mature tRNA fragments derived from tRNA^Gly^, tRNA^Glu^, tRNA^Val^, tRNA^Arg^ have been identified in human fetal liver, mouse liver and heart tissue, and various cells lines. Significantly, reduction in angiogenin levels by siRNA treatment has been shown to decrease the accumulation of tRNA^Val^ halves under stress conditions. The production of tRNA halves appears to cause translational arrest by a mechanism distinct from the better-known eIF2α dependent phosphorylation (Yamasaki et al., 2009). Of further interest, only the 5′ generated fragments are associated with translational arrest, and 5′-tiRNA^Cys^ and 5′-tiRNA^Ala^ are the most potent repressors of translation relative to other 5′-tiRNAs (Ivanov et al., 2011). The molecular mechanism of tiRNA translational repression has been linked to displacement of eIFG/A from mRNA. This proposed translational repression mechanism in mammalian cells involves the formation of a tiRNA and protein Y box binding protein 1 (YB-1) complex that associates with m^7^G cap of mRNA to inhibit eIFG/A from initiating translation (Ivanov et al., 2011).

### tRNA binding proteins during cellular stress cause disease

Translation in eukaryotes can be attenuated in response to diverse environmental stresses, including nutrient deprivation, iron limitation, protein export blockage, viral infection, UV irradiation and others. The physiological responses to these environmental challenges are coordinated by members of the eIF2α kinase family, which possess the ability to phosphorylate eIF2α when activated (Wek et al., 2006; Baird and Wek, 2012). The GCN2 kinase serves as the principal response to nutrient limitation, and reveals a special role for tRNA in its regulatory mechanism (Harding et al., 2003; Wek et al., 2006). In response to amino acid starvation, uncharged tRNA specifically activates GCN2, by virtue of binding to a HisRS-like domain. GCN2 is a serine-threonine kinase and, in addition to its kinase domain, also contains a HisRS like domain(Wek et al., 1995). The binding of tRNA to the HisRS-like domain induces a conformational change that subsequently activates the kinase domain (Dong et al., 2000; Qiu et al., 2001).

Animals lack the ability to synthesize 9 of the canonical 20 amino acids found in proteins. When an animal consumes an amino acid imbalanced diet, the deficiency in one or more essential amino acids decreases the concentration of essential amino acids in the blood stream. This elicits a short-term response, mainly controlled by the brain, and a longer-term response, largely controlled by the liver. The short term and brain specific response is based on special properties of the anterior piriform cortex, a region of the brain that functions in olfaction (Hao et al., 2005). GCN2 specifically expressed in this region acts as a special sensor of indispensible (essential) amino acids, regulating feeding behavior through its control of activating transcription factor 4 (ATF-4), the mammalian homolog of GCN4 (Harding et al., 2000; Hao et al., 2005). When rats are fed an IAA-deficient diet, the buildup of uncharged tRNA leads to activation of GCN2 (Rudell et al., 2011). This causes an adverse response, such that the animals cease feeding and search for new food sources that have a complete panel of essential amino acids. The neural circuitry for this behavior is only partially understood, but is being actively investigated (Maurin et al., 2014).

In addition to nutrient deprivation, the expression of GCN2 in the brain has recently been shown to participate in the control of long-term potentiation (LTP), a major component of synaptic plasticity associated with the enhancement of signaling between neurons. The late phase of LTP (L-LTP) is dependent on gene expression and de novo protein synthesis. A key factor in long term memory in general and LTP in particular is the repressor of cAMP responsive element binding protein (CREB), which plays a direct role in L-LTP, learning, and memory (Costa-Mattioli et al., 2005). Notably, CREB expression is negatively regulated by ATF4, which is controlled by GCN2. CREB, ATF4, and GCN2 establish a circuit in which perturbations of translational initiation lead to changes in both electrophysiological measurements of LTP and altered performance in quantitative tests of memory, such as the Morris water maze test. The expression of ATF4 is down regulated in GCN2^−/−^ animals, resulting in increased spatial memory after weak training, but poorer spatial memory after extensive training (Costa-Mattioli et al., 2005). Notably, the switch from short term to long synaptic plasticity and memory is dependent on eIF2α phosphorylation, which is a direct consequence of GCN2 activity (Costa-Mattioli et al., 2007). Collectively, these observations underscore the role of protein synthesis control in the establishment of memory. GCN2 also serves a function in proper neurobiology development and is down regulated by inhibition from imprinted and ancient gene protein homolog (IMPACT) in order to promote protein synthesis and neurite growth (Pereira et al., 2005; Roffe et al., 2013).

Recently, two reports linked inherited mutations in the gene encoding GCN2, *EIF2AK4*, with complex phenotypes arising from vascular pathology. In the first report (Eyries et al., 2014), numerous mutations in the *EIF2AK4* gene were linked to pulmonary veno-occlusive disease (PVOD), which is characterized by obstruction and blockage of pulmonary veins by fibrous, collagen rich tissue. PVOD is characterized by low diffusing capacity for carbon monoxide, septal line and lymph node enlargement, as well as occult alveolar hemorrhage (Eyries et al., 2014). By whole-exome sequencing, subjects in 13 families with the disease were found to possess multiple mutations in the *EIF2AK4* gene, in both the homozygous and compound heterozygous state (Eyries et al., 2014). Age of onset in patients varied widely from 10 to 50 years of age. Most mutations encoded stop codons in the GCN2 open reading frame, but at least two encoded missense mutations that mapped to conserved residues in the kinase domain.

In a second study (Best et al., 2014), inherited and sporadic mutations in the *EIF2AK4* gene were linked to pulmonary capillary hemangiomatosis (PCH), a progressive and eventually fatal disease characterized by capillary proliferation and invasion into alveolar septa and the bronchial wall. Symptoms included pulmonary hypertension, fatigue, cough, weight loss, and dyspnea. Exome sequencing between related individuals of patients with PCH determined that mutations in *EIFAK4* are responsible for the autosomal recessive PCH phenotype. Both affected siblings carried the same compound heterozygous mutations for which the parents were heterozygous carries, a nonsense 3438C>T (Arg1150X) mutation and frameshift 1153dupG (Val385fs) mutation (Best et al., 2014). Owing to clinical similiarity and related immunohistochemistry, PVOD is grouped with PCH, (Langleben et al., 1988; Humbert et al., 1998) and it is likely that these are different clinical description of the same basic pathophysiology. There is insufficient information to rationalize the connection between GCN2 and lung vascular pathophysiology, but an interesting hypothesis is that mutations that compromise GCN2 stress response function may expose pulmonary environment to more oxidative stress, which may predispose both mouse pulmonary hypertensive models and human subjects to pulmonary hypertension (Eyries et al., 2014).

Many studies have demonstrated the importance of GCN2 regulation during cellular stress (Grallert and Boye, 2013). In addition to its link in the pulmonary diseases described above GCN2 has an essential role in supporting tumor cell growth (Wek and Staschke, 2010) and proliferation (Ye et al., 2010, 2012). An alternative hypothesis is that GCN2 interacts with SMAD4 and SMAD1 family members, which have been implicated in signaling pathways associated with bone morphogenic protein (BMP) signaling, which is also genetically linked to pulmonary arterial hypertensive disorders (West et al., 2004). As whole genome sequencing becomes more affordable and widely available, it is likely that other clinical manifestations will be linked to altered GCN2 function, perhaps even with the neurobiological context suggested by the previously described work on nutrition sensing and LTP formation.

### The molecular biology of translation in neurons and protein synthetic machinery

Neurons possess distinct compartments, axons and dendrites collectively referred to as neurites, which are packed with specific sets of proteins to facilitate neuronal function. Positioning mRNA in these specialized compartments for protein synthesis allows neurons to remodel neurites during outgrowth, regulate synthesis of receptor subunits altering the synaptic proteome, and prevent uncontrolled recurring excitation (Turrigiano, 2011; Penn et al., 2013). A number of significant neurodegenerative diseases, such as amyotrophic lateral sclerosis (ALS), fragile X tremor ataxia syndrome (FXTAS), and myotonic dystrophy apparently involve dysfunctional messenger RNA (mRNA) localization (Ramaswami et al., 2013). Neuronal transport of mRNA from the soma to axons and dendrites is in part dependent on a family of proteins referred to as RNA binding proteins (RBPs) (Darnell, 2013). Localized translation can provide efficient increases in local protein concentrations, and regulate both immediate and delayed responses in neurons (Holt and Schuman, 2013). As our understanding of localized translation grows to include mRNA metabolism and spatial regulation, our understanding of how the other components of protein synthesis machinery are trafficked and spatially regulated in neurons should also expand.

In most cells, tRNA comprises approximately 12% of total RNA (Pace et al., 1970). Currently, tRNA molecules undergo selective transportation and localization in order for accurate protein synthesis to occur in eukaryotic cells. After transcription by RNA Pol III some tRNA molecules can be cleaved and post-transcriptionally modified in the nucleus (Hopper and Phizicky, 2003) before being transported into the cytoplasm for protein synthesis. Cytoplasmic tRNA can undergo retrograde transportation from the cytoplasm back into the nucleus (Rubio and Hopper, 2011). This mechanism may help control the rate of protein synthesis by sequestering tRNA into the nucleus during times of cellular stress. Evidence of nuclear accumulation of tRNA has been shown to occur within 15 min after glucose starvation (Whitney et al., 2007). Further indication shows that this appears to be linked to PKA activation and signaling but GCN2 pathway independent (Whitney et al., 2007). Amino acid starvation can also drive accumulation of tRNA into the nucleus (Shaheen and Hopper, 2005).

Given the emerging role of tRNA localization, it may be noteworthy that the interferon-induced tetratricopeptide repeat (IFIT) protein IFIT5 was recently shown to have tRNA binding properties (Katibah et al., 2013). IFIT5 was shown to bind RNA with 5′phosphate caps including initiator methionine iMet, tRNA^Val^, tRNA^Gly^, and tRNA^Lys^. IFIT5 appears to coimmunoprecipitate with shorter fragments rather than full-length tRNA molecules, suggesting it may help target tRNAs for degradation. IFIT5 can also bind actin, and localize to the cellular surface. In the same way that mRNA is preferentially trafficked to dendrites and axons (Wang et al., 2007), tRNA molecules may also be recruited to active sites of localized translation, setting the stage for translation to occur. Recently, single molecule studies using fluorescence *in situ* hybridization (FISH) showed that β-actin mRNA and ribosome mobilization to axons is stimulus induced, and that the β-actin mRNA becomes more available to translation machinery after synaptic stimulation (Buxbaum et al., 2014). This same group later developed a transgenic mouse with *in vivo* labeled β-actin mRNA (Park et al., 2014). β-actin mRNA appeared as part of granules and often multiple copies of the transcript were found in granules localized to dendrites. The movement of endogenous β-actin mRNA containing granules from the soma to the synapse was subsequently estimated to occur at a speed of 1.3 μm/s (Park et al., 2014). Comparably, IFT5 actin localization and tRNA binding activity could serve as a potential mechanism to inhibit localized translation, but IFIT5 localization to actin structures was shown to also be independent of RNA binding. However, there may still be potential mechanisms in place to spatially regulate tRNA localization within neurons that remain to be elucidated.

## Aminoacyl-tRNA synthetases and disease

### Overview of aminoacyl-tRNA synthetases

Aminoacyl-tRNA synthetases (ARSs) catalyze the attachment of amino acids to cognate tRNA, a key reaction that contributes to the accuracy of protein synthesis. ARSs can be divided into two distinct classes (Carter, 1993; Ibba and Soll, 2001, 2004). Class I enzymes are, with the exception of TyrRS and TrpRS, principally monomeric, contain a Rossman fold catalytic domain, and aminoacylate the 2′ hydroxyl of the terminal adenosine of their cognate tRNAs. Class II ARSs typically form dimers and tetramers, and feature a catalytic domain organized around an antiparallel beta sheet fold flanked by alpha helices. The class II enzymes couple the amino acid moiety to the 3′ hydroxyl of the tRNA's terminal adenosine. A typical animal cell contains 37 cytoplasmic and mitochondrial synthetase genes, all of which are encoded on nuclear chromosomes. Mitochondrial synthetases are given the same name as cytosolic synthetases, appended with the numeral 2 (e.g., HARS and HARS2). Collectively, they ensure accurate and efficient protein synthesis under a broad range of conditions and, significantly in multiple cellular compartments.

Pathological mutations have been identified in 10 of the genes encoding mt-ARSs (Schwenzer et al., 2013). The first of these was identified in the *DARS2* gene, which encodes mitochondrial aspartyl-tRNA synthetase (Scheper et al., 2007). Currently, pathological mutations that cause human diseases have been identified in ten genes that encode cytoplasmic tRNA-synthetases. The first cytoplasmic ARS mutation associated with a human disease, Charcot-Marie-Tooth, was discovered in glycyl-tRNA synthetase (GARS) (Antonellis et al., 2003). Here, we will focus specifically on aminoacyl tRNA synthetase mutations that affect the peripheral nervous system, sensorineural tissues, and the central nervous system (Table 2).

**Table 2:** **Aminoacyl-tRNA synthetase mutations associated with neurological disease**.

**ARS_Protein OMIM#**	**Phenotype OMIM#**	**Location of mutation(s) in protein structure**	**Phenotype**	**References**
KARS_601421	613641	Anti-codon binding domain Active site	CMTRIB	McLaughlin et al., 2010
GARS_600287	601472 600794	Dimer interface	CMT2D DHMN5A	Antonellis et al., 2003
AARS_601065	613287	Editing domain	CMT2N	Latour et al., 2010
YARS_603623	608323	Catalytic domain	DI-CMTC	Jordanova et al., 2006
HARS_142810	NC	Dimer interface	CMT	Vester et al., 2013
HARS2_600783	614926	Catalytic domain	Perrault syndrome	Pierce et al., 2011
LARS2_604544	615300	Catalytic domain	Perrault syndrome	Pierce et al., 2013
		C-terminal domain		
HARS_142810	614504	Anti-codon binding domain	Usher syndrome IIIB	Puffenberger et al., 2012
KARS_601421	613916	Anti-codon binding domain	ARNSHI	Santos-Cortez et al., 2013
DARS_603084	615281	Catalytic domain	HBSL	Taft et al., 2013
DARS2_610956	611105	Multiple	LBSL	Scheper et al., 2007
RARS2_611524	611523	Lacking exon 2	PCH	Edvardson et al., 2007
QARS_603727	ONA	Catalytic domain	MCPH	Zhang et al., 2014
		N-terminal domain		
MARS_156560	NC	C-terminal domain	HSP	Novarino et al., 2014
		N-terminal domain		
EARS2_612799	614924	Multiple	LTBL	Steenweg et al., 2012
AIMP2_600859	168600	na	Parkinson's disease	Lee et al., 2013

*NC, not confirmed; ONA, OMIM not assigned; Na, not applicable; CMT, Charcot-Marie-Tooth disease; DHMN5A, distal spinal muscular atrophy type V; ARNSHI, autosomal recessive non-syndromic hearing impairment; HBSL, hypomyelination with brain stem and spinal cord involvement and leg spasticity; LBSL, leukoencephalopathy with brainstem and spinal cord involvement and elevated lactate; PCH, pontocerebellar hypoplasia; MCPH, autosomal recessive primary microcephaly; HSP, hereditary spastic paraplegia; LTBL, leukoencephalopathy with thalamus and brainstem involvement and high lactate*.

### Aminoacyl-tRNA synthetases and peripheral neuropathies

Charcot-Marie-Tooth disease (CMT) is a hereditary peripheral neuropathy that manifests as progressive degeneration of distal motor and sensory neurons, leading ultimately to muscle weakness and atrophy of the legs and hands/arms. The various forms of CMT can be further categorized into either demyelinating type 1, which features defects in the myelin sheath surrounding peripheral nerves, and axonal type 2, which results in abnormalities in the axon of the peripheral nerve. Nerve conduction studies and electromyography (EMG) examination of patients with CMT are typically abnormal. While more than 80 genes have been linked to CMT (Timmerman et al., 2014), 30 mutations in ARS genes are associated with the intermediate and axonal autosomal dominant and intermediate autosomal recessive forms of the disease (Yao and Fox, 2013).

The first cytoplasmic ARS to be linked to CMT was glycyl-tRNA synthetase (GARS) (Antonellis et al., 2003). Since that initial report, mutations in the genes encoding four additional aminoacyl-tRNA synthetases have been linked to CMT (Wallen and Antonellis, 2013). Mutations in the *GARS* gene can cause both CMT2D and distal spinal muscular atrophy type V (DHMN5A) (Antonellis et al., 2003). It is likely significant that the synthetase enzyme encoded by the *GARS* gene is obliged to function in both the cytoplasmic and mitochondrial compartments. Similar to other class II enzymes, GARS is an obligate dimer, and several of the mutant substitutions linked to CMT alter residues located at the dimer interface (Xie et al., 2007). Some of these have been shown to either weaken or strengthen dimer formation (Xie et al., 2007). Additionally, it may also be relevant that wild type protein forms punctate structures (granules) in neurite projections, a property lost in some of the GARS CMT mutants (Antonellis et al., 2006). Other *in vitro* models of CMT GARS mutations also demonstrate a defect in localization to neurite like projections in a transfected neuroblastoma cell line (Nangle et al., 2007). It is not clear whether the phenotype is related solely to the cytoplasmic or mitochondrial distribution of GARS, or both. However, cytoplasmic ARS gene mutations associated with peripheral neuropathies (Wallen and Antonellis, 2013) indicate that dysfunctional mitochondrial protein translation is not the primary cause of these phenotypes.

A unique *Drosophila* model was created to examine the effects of the GARS CMT2D mutation on two different types of neurons, olfactory projection neurons and mushroom body γ neurons (Chihara et al., 2007). These represent two different CNS neuron populations and differ in development, morphology, and circuitry. The olfactory projection neurons showed severe defects in dendritic morphology but few axonal defects. However, mushroom body γ neurons had defects in both axon and dendritic morphology. Interestingly, as GARS is cytoplasmic and mitochondrial, individual disruption of cytoplasmic protein synthesis caused large defects in axonal and dendritic nerve end branching, while disruption of mitochondrial protein synthesis led to more dendritic nerve end branching than axonal. The *Drosophila* GARS mutations could be rescued by expression of human WT GARS. However, CMT2D mutations E71G and L129P could not rescue the defective neuronal projections and hence are loss of function mutations.

Mouse models of GARS CMT mutations have shed some light on the possible pathological mechanism for which ARSs can cause peripheral neuropathy. Interestingly, there did not appear to be significant mislocalization of GARS protein or change in GARS granule formation in the CMT2D mouse model, GARS^Nmf294/+^ (Stum et al., 2011). The GARS^Nmf294/+^ mouse model of CMT2D also showed defects in the neuromuscular junction (NMJ), reduced axon diameter, and sensory and peripheral axonal loss but no myelination defects. Importantly, this mutation does not cause a substantial loss of primary aminoacylation function (Seburn et al., 2006). More recent studies of GARS^Nmf294/+^ mice have shown that peripheral neuropathy is not caused by reduced neuronal connectivity to distal muscle fibers but that defects and denervation commonly observed at NMJs corresponds to inappropriate maturation process at distal muscle fibers (Sleigh et al., 2014).

The cytoplasmic tyrosyl-tRNA synthetase (YARS) is also linked to CMT. In particular, the mutant substitutions G41R and E196K, and deletion Δ153–156, are associated with dominant intermediate Charcot-Marie-Tooth C (DI-CMTC) (Jordanova et al., 2006). While the G41R and Δ153–156 mutations demonstrated severe catalytic defects, the E196 mutant was more active, suggesting that canonical activity defects are not responsible for the disease phenotype (Froelich and First, 2011). Furthermore, mutations are not found in domains responsible for previously reported non-canonical functions such as the cytokine activity of YARS (Wakasugi and Schimmel, 1999), suggesting that the CMT pathogenic substitutions may not be associated with YARS non-canonical activities. The mutated YARS enzyme exhibits stability *in vivo* that is comparable to the wild type enzyme (Froelich and First, 2011), but may be subject to a similar tendency to cellular mislocalization in neuroblastoma cells lines that was seen with GARS (Jordanova et al., 2006).

The bifunctional (cytoplasmic and mitochondrial) lysyl-tRNA synthetase (KARS) represents another targeted gene in CMT. One interesting CMT patient was found to be a compound heterozygote with mutations encoding both a L133H missense and a Y173SX7-frame shift mutation (McLaughlin et al., 2010). Both mutations localize to the anticodon-binding domain, however the Y173SX7-frame shift likely results in complete loss of the catalytic domain. The L113H catalytic analysis results showed a reduction in aminoacylation to levels below 25% of wild type. In addition to peripheral neuropathy, a behavioral pathology was observed in this subject. KARS is also part of the multi-synthetase complex (MSC) and can exist as either a dimer or tetramer. KARS has additional non-canonical functions involved in signaling, cell migration, and viral HIV infection, some of which are activated by post-translational modification (Motzik et al., 2013). Mitogen-activated kinase (MAPK) phosphorylation induces release of one dimer from the MSC for translocation into the nuclease, while the other dimer remains with the MSC to continue protein synthesis (Ofir-Birin et al., 2013).

Another well characterized tRNA synthetase associated with hereditary motor neuropathy is the cytosolic alanyl-tRNA synthetase (AARS). In an earlier study, characterization of the mouse strain AARS^sti^ (or “sticky” mouse) with uncharacteristic CMT phenotype, a progressive neurogeneration and a coat defect, suggested a link between these properties and an editing defect in AARS (Lee et al., 2006; Latour et al., 2010). Phenotypically, the AARS^sti^ mice have significant Purkinje cell loss and ubiquitin inclusions suggestive of protein misfolding that are absent in GARS^Nmf294/+^ mice (Stum et al., 2011). Another genetic investigation of AARS CMT2N subjects in an Australian family showed additional symptoms that included sensorineural deafness (McLaughlin et al., 2012). An attractive hypothesis is that the protein misfolding is a direct consequence of mistranslation originating from the misacylation of tRNA^Ala^ with serine instead of alanine (Guo et al., 2009).

Mutations in ARS genes have also been linked to non-CMT peripheral neuropathies. Recently, a cytoplasmic histidyl-tRNA synthetase (HARS) heterozygous mutation, R137Q, was found in a 54-year-old patient with peripheral neuropathy (Vester et al., 2013). In yeast, the mutation encoded a loss of function substitution in HARS, and the transfected mutant *HARS* gene failed to support growth. When introduced in *C. elegans*, the mutant *HARS* gene bearing the R137Q mutation caused aberrant motor neuron axonal growth and a progressive loss of motor coordination. In a fashion reminiscent of the some of the GARS-linked CMT mutants, the R137Q substitution eliminates a salt bridge at the dimer interface with D64 of the opposite monomer (Xu et al., 2012). This provides one scenario by which the R137Q substitution could bring about a loss of function, and promote the peripheral neuropathy phenotype. However, the existence of a heterozygous asymptomatic carrier with the same mutation suggests that the genetics of the system are not straightforward, and may be confounded by incomplete penetrance effects. On a related note, patients with late-onset CMT2 were identified with mutations in the methionyl-tRNA synthetase (*MARS*) gene (Gonzalez et al., 2013). The mutation affects a highly conserved arginine residue, R618C, that forms a salt-bridge interaction at the catalytic and anticodon-binding domain interface. Much like with R137Q HARS, the R618C MARS phenotype is observed to have a late onset, and does not show complete penetrance. Both mutations cause a loss of function in yeast models.

### Aminoacyl-tRNA synthetases and sensorineural diseases

Sensorineural deafness is characterized by defects in either the inner ear or the connecting auditory neural circuitry. Perrault Syndrome is described clinically as an ovarian dysgenesis with sensory hearing loss. Genetic studies indicate that mutations in mitochondrial HARS2 and LARS2 are both linked to Perrault Syndrome (Pierce et al., 2011, 2013). In the *HARS2* gene, a compound heterozygous mutation encoding both L200V and V368L is linked to Perrault Syndrome, and these substitutions alter highly conserved residues in the catalytic domain. Pyrophosphate exchange assays demonstrated that the resulting mutant proteins exhibit decreased activity, implying that the phenotype is a straightforward loss of function effect likely arising from reduced respiratory chain complex activity. Given the limitations of this single assay, the mutant substitutions may have other structural and functional consequences. Similarly, the LARS2 mutation associated with Perrault Syndrome, T522N, also targets a highly conserved residue located in the catalytic domain. By contrast, the frame shift mutation at codon 360 (c.1077delT) and T629M compound heterozygous mutations found in another Perrault syndrome patient localizes to the poorly conserved leucine-specific C-terminal domain. Yeast complementation assays showed that T522N LARS2 did not support growth, while T629M LARS2 did. Notably, *C. elegans* models carrying the mutations were completely sterile, recapitulating at least one component of the disease phenotype.

Cytoplasmic HARS has been also been associated with a heterogeneous sensorineural disease that causes deaf-blindness Usher Syndrome Type IIIB. Usher Syndrome is a polygenetic disease with at least 11 different loci identified. Most of the genes associated with Usher Syndrome are typically involved in inner ear hair cell morphology and development (Yan and Liu, 2010). The three clinical subtypes are divided up by severity of the symptoms and age of onset that the phenotype presents. A homozygous mutation in HARS, Y454S, was discovered in patients with Usher Syndrome IIIB (Puffenberger et al., 2012) and is the first cytoplasmic HARS mutation associated with disease. In these patients hearing and vision are severely impaired early in childhood, and fever induced hallucinations can occur. While peripheral nerve function seems to be normal, there is some mild trunk ataxia. The Y454S mutation, which is localized specifically on the surface of the anticodon-binding domain (ABD), juxtaposes the catalytic domain of the second monomer. Y454 hydrogen-bonds with residue E439 within the anticodon binding domain, and E439 is positioned to interact with K148 from the catalytic domain of the second monomer to form a salt-bridge interaction (Xu et al., 2012)^1^. Initial aminoacylation data employing wild type and mutant versions of the mouse enzyme demonstrates that the Y454S substitution is unlikely to be a simple loss of function. Accordingly, current efforts are testing the hypothesis that HARS has a secondary function that is impaired by the Y454S substitution.

Hearing loss phenotypes have been linked to a number of ARSs in addition to HARS. Patients in three unrelated consanguineous Pakistani families suffered from an autosomal recessive non-syndromic hearing impairment (ARNSHI) linked to mutations in KARS in the known ARNSHI-associated locus DFNB89 (Santos-Cortez et al., 2013). One of the mutations is predicted to encode an Y173H substitution in a residue located in the oligomer-binding (OB) fold motif of the KARS anticodon binding domain. In this location, the substitution could negatively affect tRNA binding and/or catalysis. Significantly, the mouse organ of Corti and vestibular system features many cell types, including inner/outer hair cells spiral ligament, and sulcus and spiral limbus cells of the vestibular membrane epithelium where KARS is prominently expressed.

### Aminoacyl-tRNA synthetases and central nervous system diseases

In contrast to the peripheral nervous system and the sensorinervous system, there are few reports linking ARSs to central nervous system diseases, which often affect specific brain regions. The autosomal recessive monogenetic disease Leukoencephalopathy with Brain stem and Spinal cord involvement and elevated Lactate (LBSL) represents the first well-characterized CNS disease associated with an ARS. Genetic analysis of some 30 different families helped link the disease to mutations in the *DARS2* gene encoding mitochondrial AspRS (Scheper et al., 2007). Hallmarks of the disease defined by MRI imaging include abnormalities of the white matter in the cerebellum, spinal cord, and brainstem. While the mutant substitutions in LBSL are predicted to impair dimer formation and decrease DARS2 catalytic function, mitochondrial respiratory chain complex activity in these LBSL patients was normal (Scheper et al., 2007).

Mutations in the *DARS* gene encoding cytoplasmic AspRS were identified in patients with hypomyelination with brain stem and spinal cord involvement and leg spasticity (HBSL), an inherited white matter disease (Taft et al., 2013). These DARS coding changes may affect enzyme activity by either disrupting tRNA binding or reducing protein expression (Van Berge et al., 2012, 2013). Interestingly, patients with the DARS-linked white matter disease exhibited the same types of white matter abnormalities in brain stem and spinal cord regions by MRI imaging that were observed in patients with the DARS2 mutations linked to LBSL. Cases of white matter disease associated with ARSs are not exclusively restricted to AspRS. A recent report described a disease called leukoencephalopathy with thalamus and brainstem involvement and high lactate (LTBL) linked to mutations encoding mitochondrial glutamyl-tRNA synthetase (EARS2) (Steenweg et al., 2012). The full characterization of the role of ARS function in these diseases is at an early stage, and it is too soon to conclude that they are entirely explained by the sensitivity of neurons to decreased output in translation.

Several other relatively recent reports highlight the potential association of mutations in ARS genes with neurodegenerative diseases. For example, the lethal heterogeneous neurodegenerative disease pontocerebellar hypoplasia (PCH6) was linked to the *RARS2* (mitochondrial arginyl-tRNA synthetase) gene in a patient with a homozygous frameshift mutation predicted to generate a truncated protein (Edvardson et al., 2007). Other case studies of PCH6 subjects subsequently identified additional *RARS2* mutations (Glamuzina et al., 2012). In the zebrafish model, RARS2 is highly expressed in the brain 24 h post fertilization. Of further interest, zebrafish knockdown models of TSEN54 (subunit associated with RNA splicing previously described) and RARS2 produce comparable phenotypes characterized by brain hypoplasia and increased cell death, but little effect on brain patterning. These interesting results suggest there may be a common pathological PCH phenotype associated with loss of function of the *TSEN* and *RARS2* alleles, as well as a demand for specific spliced tRNAs products at specified times during neuronal development (Kasher et al., 2011). Recently, whole-exome sequencing identified that *QARS* is a causative gene in affected individuals of the two families with children affected by autosomal-recessive primary microcephaly (MCPH) (Zhang et al., 2014). Symptoms of this disease are associated with intellectual disability, seizures during infancy, and atrophying in brain regions of the cerebellar vermis and cerebral cortex. This disease phenotype is similar to the PCH caused by mutations in RARS and TSEN complex previously described. However, specific brain regions are differentially affected for each disease. Four variants were identified, two of which were localized to the catalytic domain and the remaining two to the tRNA binding N-terminal domain. Activity studies showed that all mutations caused a loss of functional protein. Also, a zebrafish model demonstrated that QARS is essential for proper eye and brain development.

The potential value of the zebrafish in studying ARS-linked neurodegenerative diseases is suggested by another recent report in which methionyl-tRNA synthetase (*MARS*) was identified in an exome sequencing study as one of 15 genes linked to hereditary spastic paraplegias (HSP) (Novarino et al., 2014). HSP is characterized by the degeneration and progressive loss of corticospinal motor neuron tract function; patients typically present with lower limb spasticity, seizures, ataxia, peripheral neuropathy, intellectual disability, skin, and visual defects. The potential roles of many of the genes reported in this study in HSP were validated in the zebrafish model; the MARS mutation was too severe to be fully evaluated.

Another important connection between ARS function and neurodegenerative disease reported recently is the linkage between the aminoacyl-tRNA synthetase complex interacting multifunctional protein-2 (AIMP2, also referred to as p38) and familial Parkinson's disease. Initially, AIMP2 was reported to be deposited in Lewy bodies, and its accumulation had been noted in some familial cases of Parkinson's disease (Corti et al., 2003; Ko et al., 2005). Later, AIMP2 was determined to be a substrate of the E3 ligase PARKIN. Multiple loss of function mutations in the gene encoding PARKIN are a common cause of familial Parkinson's disease. While the complete basis of AIMP2-linked neurological pathophysiology is not yet clear, it is possible that an important secondary role of AIMP2 is modulating neuronal protein turnover; when this process is dysfunctional, particular cell death pathways (e.g., “parthanatos”) may become activated.

## Future prospects for the role of tRNA in human disease: the role of spatial and temporal control of tRNA and the translational machine in disease pathophysiology

Given the emerging role of mRNA transport and localization in neuronal function (Wang et al., 2007), it would not be surprising if the temporal and spatial regulation of aminoacyl-tRNA synthetases in neuronal compartments such as axons and dendrites were not similarly important. Protein synthesis in these diverse compartments clearly requires the full translational apparatus in addition to the messenger RNA. At present, this remains an underexplored area of tRNA and ARS biology, and a role for tRNA and ARS in localized translation in neurons should be investigated. Some of the models seeking to explain the link between the ARS and CMT have proposed that the mutant substitutions create localization defects for the affected enzymes. As yet, the evidence is not definitive, and the cellular mechanisms that traffic and localize these enzymes are essentially unknown. Is the localization of ARS enzymes to distal sites of the axon and dendrites dependent on vesicular transport, tRNA, or other trafficking proteins? In addition to the well-known Multi-Synthetase Complex (MSC), with what types of protein complexes are the ARS enzymes associated, and what positions them at the site of local translation in dendrites and neurons? Are there signaling pathways that are necessary to initiate localization of ARS enzymes to these regions? Furthermore, there may be mechanisms in place (e.g., post- translational control) to spatially regulate the activity of ARS such that tRNA is not aminoacylated until the proper cues are received to synthesize proteins. These questions, and other issues related to functional compartmentalization, should keep the role of tRNA and ARS in complex human diseases as a major question for years to come.

### Conflict of interest statement

The authors declare that the research was conducted in the absence of any commercial or financial relationships that could be construed as a potential conflict of interest.
